# Multisensory Evaluation of Muscle Activity and Human Manipulability during Upper Limb Motor Tasks

**DOI:** 10.3390/bios13070697

**Published:** 2023-06-30

**Authors:** Jose M. Lopez-Castellanos, Jose L. Ramon, Jorge Pomares, Gabriel J. Garcia, Andres Ubeda

**Affiliations:** 1 Human Robotics Group, University of Alicante, 03690 San Vicente del Raspeig, Spain; jl.ramon@ua.es (J.L.R.); jpomares@ua.es (J.P.); gjgg@ua.es (G.J.G.); andres.ubeda@ua.es (A.U.); 2 Department of Systems Engineering, National Autonomous University of Honduras, Tegucigalpa 11101, Honduras

**Keywords:** human manipulability, electromyography, upper limb, rehabilitation

## Abstract

In this work, we evaluate the relationship between human manipulability indices obtained from motion sensing cameras and a variety of muscular factors extracted from surface electromyography (sEMG) signals from the upper limb during specific movements that include the shoulder, elbow and wrist joints. The results show specific links between upper limb movements and manipulability, revealing that extreme poses show less manipulability, i.e., when the arms are fully extended or fully flexed. However, there is not a clear correlation between the sEMG signals’ average activity and manipulability factors, which suggests that muscular activity is, at least, only indirectly related to human pose singularities. A possible means to infer these correlations, if any, would be the use of advanced deep learning techniques. We also analyze a set of EMG metrics that give insights into how muscular effort is distributed during the exercises. This set of metrics could be used to obtain good indicators for the quantitative evaluation of sequences of movements according to the milestones of a rehabilitation therapy or to plan more ergonomic and bearable movement phases in a working task.

## 1. Introduction

The study of human movement has important implications in both professional and rehabilitation activities that imply the use of the upper limbs. Indeed, the precise evaluation of arm kinematics in manipulation tasks has been applied to the study of ergonomics in many previous works [1,2,3]. There is a large number of published studies that describe the kinematic analysis of human movement in a variety of working tasks, e.g., in sewing [4], hammering [5] or material handling [6]. On the other hand, applications to motor rehabilitation have been explored in robot-assisted contexts [7,8] or for the monitoring of patient movement [9,10,11].

One interesting metric to evaluate human upper limb movements is manipulability. In the context of robotic manipulation, manipulability has been proposed as a quality metric to infer the manipulating ability of robotic mechanisms in positioning and orienting end effectors [12]. In other words, it measures the capacity to change the end effector’s pose as a function of the joint configuration. This index can be translated to a human context with the so-called human muscular manipulability (HMM) [13]. In this case, the metric accounts for the forces that are generated by the muscles acting on the arm joints and can be addressed through some indices, such as the Kinematic Manipulability Index (KMI). Another relevant index of movement comfort is the Local Conditioning Index (LCI), which serves as a dexterity metric indicating how close the robotic manipulator is to a singularity [14]. A robot singularity is a configuration in which the robot end effector becomes blocked in certain directions, so, in the case of human movement, a singularity accounts for extreme positions where limb joints are limited in movement. In this work, we also apply the LCI index to the human context.

The combination of manipulability indices with muscular information can be of great interest to study ergonomic aspects in both clinical and working environments. Measuring muscular fatigue, effort or coordination in a working task is critical to prevent future musculoskeletal disorders, while the evaluation of motor function during rehabilitation activities provides a tool for the monitoring of the evolution of a patient’s recovery and the readaptation of the therapy in a more effective manner. Multiple studies have highlighted the possibility of using surface electromyography to measure the electrical activation of the muscles to evaluate motor performance in a rehabilitation context [15,16,17]. Working activities can also benefit from the monitoring of muscular activity [18]. In this case, most of the studies are centered on evaluating muscle fatigue during upper limb activities such as manual lifting [19], and they focus less on motor coordination aspects.

In this work, we evaluate the relationship between manipulability indices (KMI and LCI) and a variety of muscular factors of the upper limb during specific movements that include the shoulder, elbow and wrist joints. For this purpose, we employ different types of inputs, including motion sensing cameras and sEMG electrodes.

## 2. Materials and Methods

### 2.1. Subjects

Nineteen healthy subjects (3 women and 16 men) participated in this study (age 26.89±7.9 years, height 175±7 cm and weight 76±13.9 kg). All participants were right-handed and had no known neuromuscular or sensory disorders. Before the development of the experiment, the participants were informed about the nature of the exercises and gave their informed consent. The study was conducted in accordance with the Declaration of Helsinki.

### 2.2. Setup and Equipment

**Figure 1:**
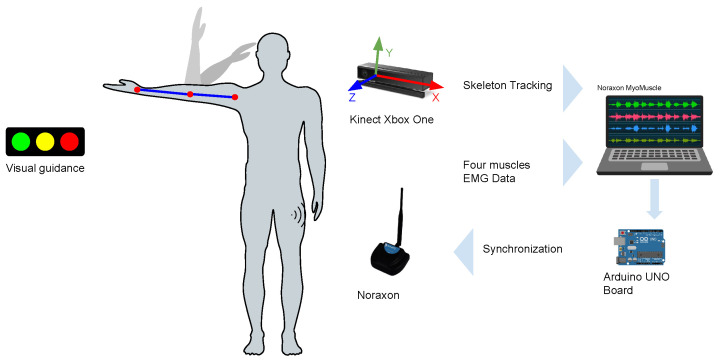
Setup to capture participant data.

The general setup in this study is depicted in Figure 1. The setup consisted of a Kinect v2 camera, a PC, an Arduino UNO and a Noraxon MiniDTS system.

The Kinect has a frequency of 30 Hz and was used for the skeleton tracking of the participant. The Arduino UNO was utilized to send a sync trigger from the PC to the Noraxon software, indicating the beginning of data collection for post-processing tasks. The Noraxon mini DTS system consists of two main components, a mini DTS receiver and four single-channel DTS EMG wireless sensors, with a selected sample rate of 1500 Hz in this case. The values captured by each sensor are sent to the DTS receiver using radio frequencies between 2.4 and 2.5 GHz, in a short-range network. The sEMG sensors have an input range of ±5 mV and internally apply a first-order high-pass filter set to 10 ± 2 Hz. Further details are listed in Table 1.

The Noraxon MyoMuscle software was used to record the information from the EMG sensors, each one connected to two disposable disc surface electrodes with approximately 1 cm of separation center-to-center. The PC was running a custom C# code to store the captured information with the Kinect camera.

### 2.3. EMG Sensor Placement and MVC Procedures

The electrodes were placed along the muscle fibers on the flexor carpi ulnaris (FCU), extensor carpi ulnaris (ECU), biceps brachii (BB) and deltoideus medius (DM). Prior to the placement of the electrodes, the skin area was cleaned using alcohol and a light abrasive gel was applied.

Once the electrodes were placed on the forearm and the location was validated, the maximum voluntary contraction (MVC) of each muscle was recorded. The participant was asked to perform the following movements for 3 s, and the maximum value of the registered sEMG was taken as the MVC value. In the case of the FCU, the participant was asked to place the forearm in pronation on a table, with an extended hand, and to perform a wrist extension, while the assistant applied force on the hand of the participant to generate resistance. Regarding the ECU, the participant was asked to place the forearm in supination on a table, with an extended hand, and then execute a wrist flexion, while the assistant exerted force downwards on the hand of the participant. As for the BB, while the participant was seated, they were asked to place the palm of their hand under the table and exert force upwards, while the assistant applied force downwards on the table. In the case of the DM, resistance was achieved using elastic bands. The participant was asked to perform an arm abduction against resistance, using the back of the hand to extend the bands.

### 2.4. Exercises and Data Acquisition

The experiment consisted of two exercises, with ten repetitions for each one, with a total of 20 repetitions per participant and approximately 25 min in total duration. The two proposed upper limb tasks integrated activities derived from exercises that were composed of a series of active and passive upper limb rehabilitation tasks for stroke patients [20]. The phases of the exercises were formulated in order to determine the manipulability indices related to extreme positions of the arm and joint angle variations. A priori, the relationship between the participant’s muscle activity during these exercises and the manipulability indices was not known.

Prior to each exercise, the participant had to stand on two markings on the floor, in front of the RGBD camera, with arms in a relaxed position. A simple visual guideline to indicate the duration of the rest and trial activity period was provided with the aid of a smartphone, situated on a table in front of the participant. When the red color was visible, it indicated the resting period, and when the green was visible, it indicated the activity period (see Figure 1).

**Figure 2:**
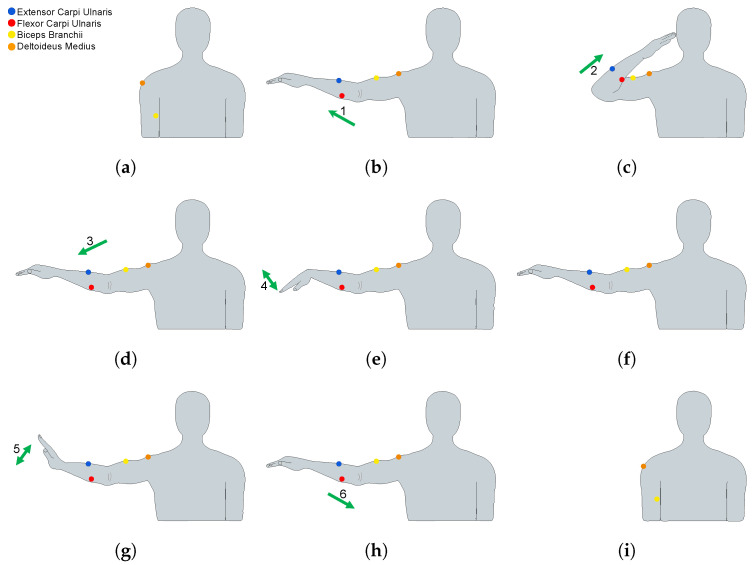
(**a**–**i**) Movements performed in exercise 1 (frontal plane). The number of phases of the exercises is indicated.

The first exercise (Figure 2) consisted of performing the following movement phases: the participant started in a resting position, and then the participant had to perform an arm abduction (phase 1) approximately at 90 degrees. An elbow flexion (phase 2) followed (until almost touching the forehead with the tip of the fingers); subsequently, an elbow extension (phase 3) movement was performed, followed by a wrist flexion with extended hand (phase 4), and then they returned the wrist and the hand to a neutral position. Then, they performed a wrist extension with extended hand (phase 5) with palm facing downwards and returned the wrist and the hand to a neutral position; finally, the participant placed the arm in a resting position (phase 6).

**Figure 3:**
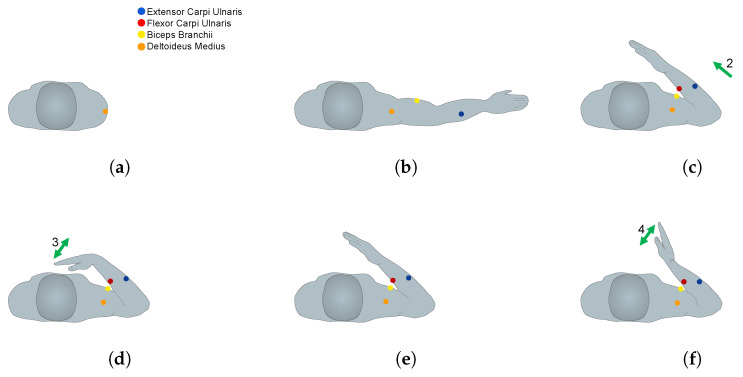

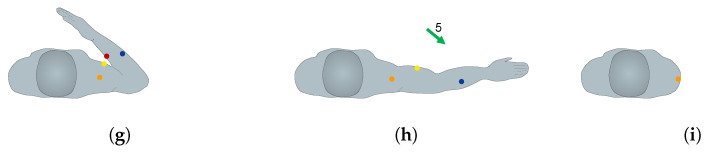
(**a**–**i**) Movements performed in exercise 2 (transverse plane). The number of phases of the exercises is indicated.

The second exercise (Figure 3) consisted of performing the following movement phases: as in exercise 1, the participant started at a resting position, and then they had to perform an arm abduction (phase 1) movement at approximately 90 degrees. Then, they executed an elbow flexion movement towards the chest (phase 2), performed a wrist flexion with extended hand and returned the wrist and the hand to a neutral position (phase 3). This was followed by a wrist extension with extended hand and then they returned the wrist and the hand to a neutral position (phase 4). Finally, the participant performed an elbow extension (phase 5) and then returned the arm to the resting position.

## 3. Data Analysis

### 3.1. Human Arm Kinematic Model

**Figure 4:**
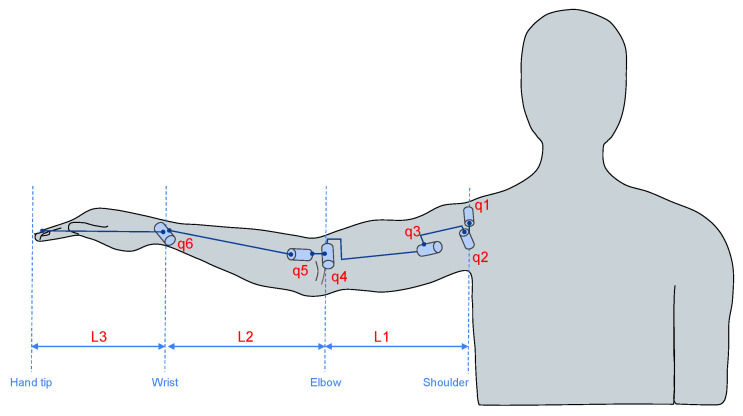
Human arm modeled as a three-segment mechanism.

To calculate the HMM, the arm musculoskeletal system was modeled as a plane series mechanism of three segments, where the represented joints were the shoulder, elbow and wrist. This model had six degrees of freedom (DOF) as follows: shoulder abduction/adduction (q1), shoulder flexion/extension (q2), shoulder rotation (q3), elbow flexion/extension (q4), pronation/supination (q5) and wrist flexion/extension (q6). L1, L2 and L3 are the arm, forearm and hand length, respectively. See Figure 4 for details.

### 3.2. Human Manipulability

The HMM is a kinematic approach to evaluating the dexterous manipulability of a joint mechanism in the human context, i.e., the human arm. This measure describes the relationship between joints and limb endpoints in terms of velocity, acceleration or force applied by the mechanism. This concept can be depicted by an ellipsoid around the endpoint of the mechanism to represent the optimal velocity, acceleration or force capacity in the ellipsoid axes’ direction.

To compute the manipulability as defined by Yoshikawa [21], the capacity of positioning of a manipulator is measured with a scalar value given by

(1) $$w=\sqrt{det[JJ^{T}]}$$

where *J* is the Jacobian matrix and *w* represents the KMI.

Following this approach, the human arm was modeled in Matlab (R2021a, Natick, MA, USA) using the parameters of the Denavit Hartenberg (DH) representation [22] by means of a robotic toolbox [23]. For each user, the values of the joint angles and lengths were taken from the skeleton points captured with the Kinect camera. The whole kinematics of the right arm defined using the DH parameters is listed in Table 2.

There are other parameters that can be used to obtain a good and intuitive measure of the dexterity of the joint mechanism in grasping or manipulating tasks, namely the dexterity index (Id) and the LCI. The dexterity index provides a measure of how comfortably the joint mechanism is working, i.e., the motion capability of the joint mechanism in the workspace, and could give different values depending on the joint position. On the other hand, the LCI yields a value between 0 and 1; a value of 0 indicates that the joint mechanism is closer to a singularity and a value of 1 suggests that it is far from a singularity [14].

(2) $$I_{d}=∥J∥\;\cdot \;∥J^{-1}∥$$

(3) $$LCI=I_{d}{\left(J\right)}^{-1}$$

Using the arm joint angles and lengths between joints to build the model, and the Jacobian matrix of the manipulator, the KMI and the LCI were computed applying Equations (1) and (3), respectively.

### 3.3. Data Synchronization

Before further processing, a set of markers were manually placed in each sEMG signal file using the software MyoMuscle of Noraxon, to indicate the beginning and end of the muscle activation during each trial of the exercise; this information was added to the data file in a separate channel indicating the time that the markers were placed. The data file was exported as a Matlab compatible file. The processing of participants’ data was performed in Matlab using custom code. For initial processing, each raw sEMG signal was segmented using the trigger marks received via the Arduino UNO, indicating the starting and ending points of the whole exercises; then, the data of the manipulability indices were resampled to the same length as the sEMG signals.

### 3.4. Analysis of EMG Factors

To compute the metrics on the sEMG signals for exercises 1 and 2, two approaches were followed. The first one consisted of calculating the mean and standard deviation per participant, as every channel comprised ten filtered and rectified muscle activations. The sEMG signals were passed through an 8th-order Butterworth bandpass filter (20–300 Hz), rectified and smoothed using a median filter window of 3500 bins in order to obtain the envelope of the signal. Then, each signal was normalized according to the recorded MVC for each participant’s muscle. The muscle’s activity during trials was obtained using the markers set manually, and then the average duration of the activity was computed and all the trials’ activities were resampled in order to ensure the same length. For the sEMG signal results, the mean and standard deviation of the KMI and LCI were computed using the same intervals as for the sEMG signals. The beginning and end of the different phases of the exercises were represented with vertical blue lines and the number of the phase of the exercise. After obtaining the values for each participant, the mean and standard deviation across all participants were obtained.

The second approach is to calculate the root mean square (RMS), mean absolute value (MAV), wavelength (WL) and sign slope change (SSC) on the raw sEMG (see Equations (4)–(7)). The RMS represents the mean signal magnitude and gives an insight into the muscle activation. The MAV gives the area under the rectified sEMG signal, i.e., the signal amplitude over a period of time. The WL is the sum of the distances between samples in the window (i.e., the muscle activity during a trial) and gives a measure of the waveform amplitude, frequency and duration over a period of time. Finally, the SSC is used to obtain frequency information from the sEMG by counting the number of times that the slope sign changes and is performed using a threshold function to avoid background noise.

(4) $$RMS=\sqrt{\frac{1}{L}\sum_{i=1}^{L}\overset{2}{\underset{i}{x}}}$$

(5) $$MAV=\frac{1}{L}\sum_{i=1}^{L}\left|x_{i}\right|$$

(6) $$WL=\sum_{i=1}^{L-1}\left|x_{i+1}-x_{i}\right|$$

(7) $$SSC=\sum_{i=2}^{L-1}f\left[\left(x_{i}-x_{i-1}\right)\times \left(x_{i}-x_{i+1}\right)\right]$$

$$f\left(x\right)=\left\{\begin{array}{cc} 1 & \mathrm{if}\;x\geq \mathrm{threshold}\\ 0 & \mathrm{Otherwise} \end{array}\right.$$

where *L* is the length of the muscle activity data and $x_{i}$ denotes the amplitude value of the sample *i*. To compute SSC, 100 μV was used as a threshold value. First, the sEMG signals were resampled to the mean length of the exercise trial duration across all participants, and then the previous metrics were computed on each section or phase of the exercises along each participant’s muscle activity. Finally, the mean and standard deviation across all participants were obtained. The duration of the exercises was depicted using vertical blue lines and was approximated as the mean duration of each exercise phase across all participants. The second part consisted of computing the metrics on every instance of muscle activity during the exercise trials along the channel data of every participant, obtaining the mean and standard deviation per user muscle, and finally the results across all the participants were calculated.

### 3.5. Statistical Analysis

The statistical analysis was performed on the data using Matlab. The Wilcoxon sum rank test with significance of α=0.05 and Bonferroni post-hoc tests were used.

## 4. Results

### 4.1. EMG and Manipulability

**Figure 5:**
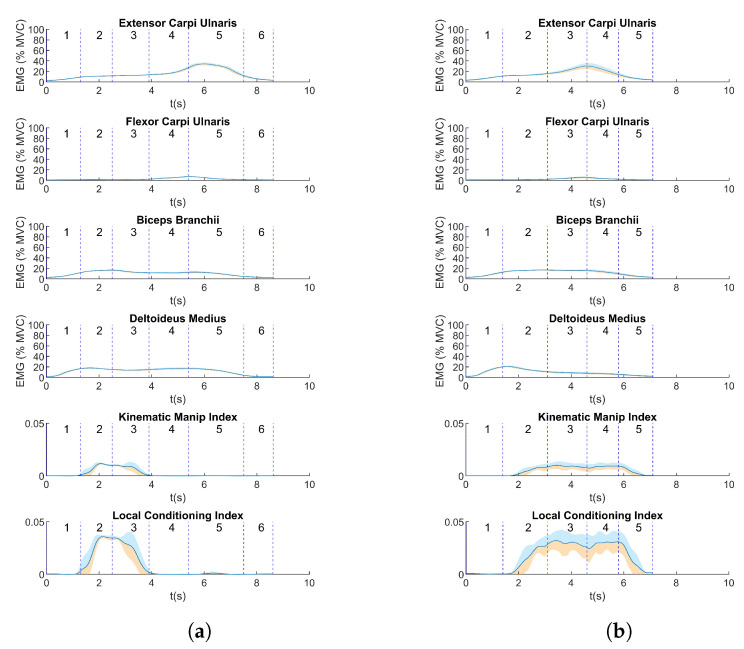
(Mean ± sd) Muscle activity and manipulability indices for (**a**) exercise 1 and (**b**) for exercise 2 for a representative participant.

The mean EMG values and manipulability indices of a representative participant are shown in Figure 5a. For exercise 1, the highest values of the KMI and LCI were obtained almost at the beginning of the exercise, with a combination of high activation of the DM and BB, corresponding to phases 2 and 3 of the trial (elbow flexion and extension). As soon as the BB activation started to decrease, the KMI and LCI dropped to a small value. The DM remained active until the return of the arm to a resting position. Regarding the FCU and ECU muscles, when each of them reached the peak activity, both indices remained closer to zero. On the other hand, for exercise 2, the relation between the muscle activity and manipulability indices was not so evident compared to the peaks in muscle activity in exercise 1. On the contrary, similar values of the indices were observed over a longer period than in exercise 1 (Figure 5b).

**Figure 6:**
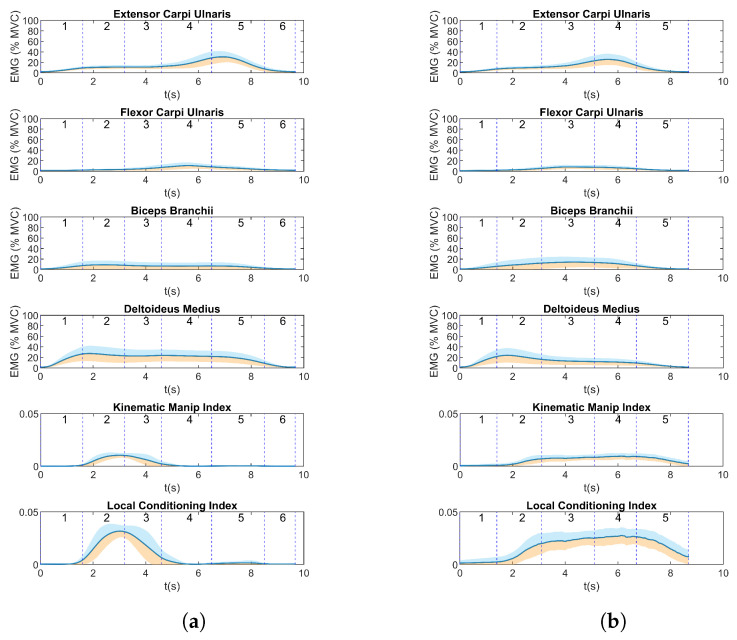
(Mean values ± sd) Muscle activity during execution of trials for (**a**) exercise 1 and (**b**) exercise 2, for all participants. The blue lines indicate the beginning or end of the phases of the proposed exercises as depicted in Figure 2 for exercise 1 and in Figure 3 for exercise 2.

This behavior was shared across all participants (Figure 6). For exercise 1, the mean KMI had a peak at the beginning of the exercise, showing that, for this activity, all the participants obtained a high value of this index during phases 2 and 3 of exercise 1; for the subsequent phases, the value decreased to a zero value or closer to zero. Regarding the mean activity of muscles and indices for exercise 2, as well as the particular case shown previously in Figure 5, in general, the mean KMI was observed after phase 1 and maintained similar values throughout the execution of the other phases.

### 4.2. EMG Metrics

**Figure 7:**
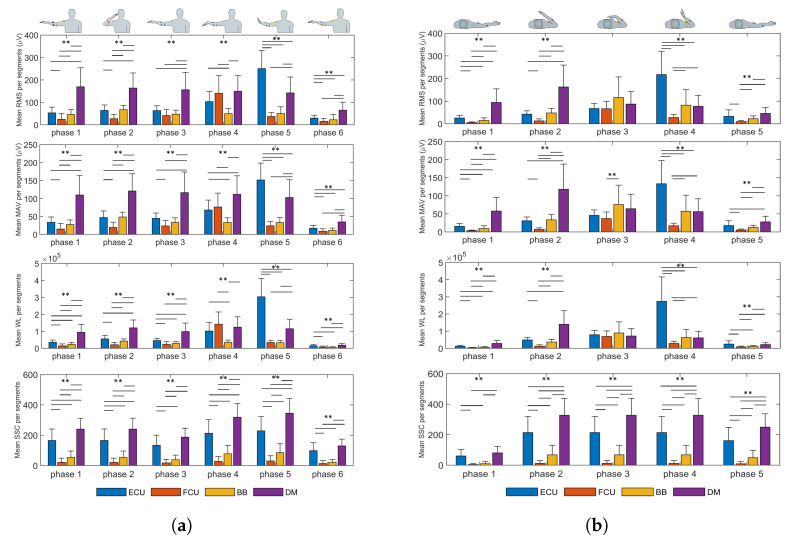
(Mean values and sd) EMG metrics for (**a**) exercise 1 and (**b**) exercise 2, for all participants, computed on muscle activity during the trial phases. The double-asterisk indicates statistically significant difference (p<0.01).

Figure 7 shows the results of the metrics computed (mean ± sd) for every phase of the proposed exercises, indicating statistical differences between muscles. With respect to the RMS, in exercise 1, the DM was more active during the whole exercise, and its activation was higher than that of the other muscles in phases 2 (163.76 ± 67.27 μV, p<0.008) and 3 (156.30 ± 77.55 μV, p<0.008), which corresponded to arm abduction. BB showed similar activity for the whole exercise, with no particularly high activation in any phase. In phase 4, the difference between the ECU, FCU and DM was not statistically different. Regarding phase 5, the ECU showed higher muscle activation during the whole exercise (250.63 ± 81.48 μV, p<0.008). In the case of exercise 2, the ECU again showed the highest activation, but in phase 4, which comprised, similarly to exercise 1, a wrist extension (217.78 ± 102.83 μV, p<0.008), none of the muscles showed constant high activation throughout the whole task.

In reference to the results for MAV computation, in exercise 1, the mean activation of DM was higher in phase 1 (109.89 ± 53.92 μV, p<0.008), phase 2 (121.02 ± 48.09 μV, p<0.008), phase 3 (116.45 ± 57.58 μV, p<0.008) and phase 6 (34.97 ± 18.58 μV, p<0.008), which correspond to arm abduction, elbow flexion, wrist flexion and arm adduction. In phase 5, the ECU showed higher mean activation than in the other phases (151.69 ± 47.10 μV, p<0.008), which was expected because the phase comprised wrist extension. In exercise 2, the scenario was very similar to that of the RMS results.

In the case of the WL results, these were similar to those obtained for the MAV and RMS in both exercises, as the WL also accounts for muscle activation over time. It can be observed that in exercise 1, the DM produced higher values in all phases, but not in phase 5, when the activation of the ECU was higher (3.0362 × 10^5^ ± 1.0757 × 10^5^, p<0.008) due to the wrist extension movement, as in phase 4 of exercise 2 (2.7398 × 10^5^ ± 1.4168 × 10^5^, p<0.008).

Regarding the values of SSC for exercises 1 and 2, the DM showed higher results in both exercises, indicating that it was the most active muscle, even though, compared to some other muscles, it showed lower amplitudes in some phases. The ECU had the second-highest values across all phases, showing that it was strongly activated in both tasks. The BB showed uniform values in exercise 2 during phases 2–4, when the elbow flexion was maintained.

**Figure 8:**
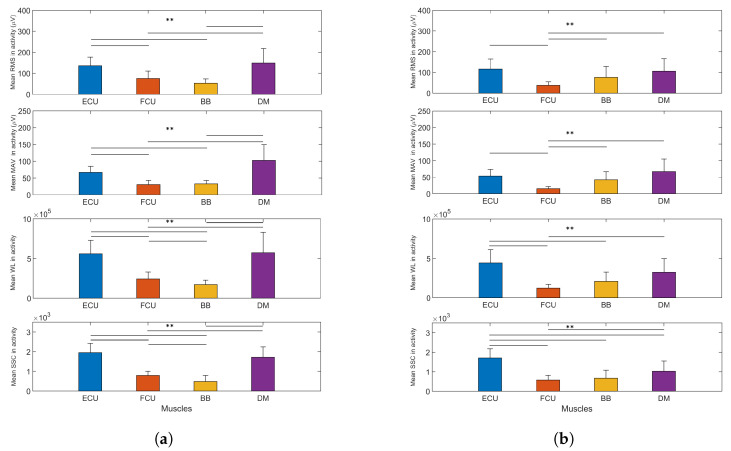
(Mean values and sd) EMG metrics for (**a**) exercise 1 and (**b**) exercise 2, for all participants, computed on muscle activity during the whole task. The double-asterisk indicates statistically significant difference (p<0.01).

The metrics were also computed on the muscle activity across the whole exercise (see Figure 8). For the RMS, the muscle activity of the DM (149.13 ± 68.85, p<0.008) was significantly higher than that of the FCU and BB in exercise 1; the same occurred between the ECU (136.01 ± 40.76, p<0.008) and FCU, as well as BB. In exercise 2, the differences between the DM (105.59 ± 60.13, p<0.008), ECU (115.79 ± 48.49, p<0.008) and BB (76.46 ± 52.68, p<0.008) with respect to the FCU were statistically significant. Regarding the MAV, the DM produced the highest mean activity level during both exercises, but only the differences versus FCU and BB were statistically significant for exercise 1, and with respect to the FCU in exercise 2. In both the RMS and MAV, it was shown that the BB was more activated in exercise 2 than in exercise 1. With respect to the WL, in exercise 1, it also could be observed that the DM reached higher levels across the whole task than the FCU and BB (5.7189 × 10^5^ ± 2.5918 × 10^5^ p<0.008), but there was no significant difference compared to the ECU. In the case of exercise 2, the ECU yielded a significantly higher value than the FCU and BB (4.4055 × 10^5^ ± 1.6818 × 10^5^, p<0.008) because of its high amplitude in wrist extension. In regard to the SSC, for exercise 1, there was no significant difference between the ECU and DM, but the rest of the differences were statistically significant. In exercise 2, the value reached by the ECU was significantly higher than those of the other muscles, including the DM.

## 5. Discussion

Considering the values shown in Figure 6, the movements performed in exercise 1 seem appropriate to determine which motor tasks produce an effect in manipulability indices. In this set of exercises, the peak in the KMI, as in the LCI, was produced after the participant had performed an elbow flexion movement (phase 2) and before the elbow extension movement (phase 3). Moreover, movements such as wrist flexion and extension produced manipulability indices closer to zero in this phase of the exercise, meaning that these arm positions could represent joint configurations that are closer to a singularity or are more limiting regarding freedom of movement due to being in a low-manipulability zone [24]. The values of manipulability obtained in this study are consistent with those found in [25].

In addition, in Figure 6, it can be observed that the manipulability indices, in contrast to the results for exercise 1, did not have a single peak but higher values were obtained in exercise 2, for almost the entire duration of the exercise. Moreover, the manipulability indices started increasing after the beginning of phase 2, when elbow flexion started, while the DM and BB muscles were activated almost throughout the whole test. As in exercise 1, the values close to zero in both the KMI and LCI at the beginning of the exercise suggest limitations in the range of movement due to the arm position. In both exercises, the movement involved in the phases of high-manipulability indices was elbow flexion, as observed in Figure 6 (phase 2 in exercise 1 and phases 2–5 in exercise 2), but there was not an evident relation between the muscle activity and the manipulability output. In both exercises, the activity of the BB was lower than for the other muscles. In addition, wrist flexion and wrist extension seemed to not have a major contribution to the manipulability index, and this was more evident in exercise 1. The BB was activated more when participants performed wrist flexion (phase 3) than in phase 4 of exercise 1, when the activation of the DM decreased. Moreover, the BB and DM supported movement at the endpoint of the arm, when the FCU performed a contraction in exercise 2.

The analysis of the EMG signals through different metrics, along with the participant’s arm motion capability, could be helpful to identify muscle effort and simplify exercise sequences accordingly. This could be important to assess participants’ progress in activities such as physical therapy, but also to evaluate working loads in tasks that require arm lifting. However, their behavior does not have a clear correlation with manipulability indices. For exercise 1, high manipulability was present in phases 2 and 3 (mainly), while the EMG parameters showed low values in general. Peaks in most of the metrics were more visible in the last few phases of the exercise and especially significant in particular muscles, such as the DM, corresponding to low manipulability. This behavior was very similar across all metrics. On the contrary, in exercise 2, a clear peak in ECU muscle activity for RMS, MAV and WL was present in phase 4, where the manipulability indices were higher. This apparent contradiction suggests that a relationship, if significant, between EMG metrics and manipulability is difficult to deduce from the current study. A possible means of establishing correlations is to introduce a deep learning analysis of the EMG metrics to infer manipulability. This deeper evaluation could shed light on the possible link between a comfortable upper limb pose and specific muscle activation. This would be useful to track, for instance, comfort in a proposed set of exercises or motor tasks, being good indicators for the quantitative evaluation of sequences of movements according to the milestones of rehabilitation therapies or to plan more ergonomic and bearable movement phases in a working task. Another possible approach implies the fusion of both sets of metrics (EMG and manipulability) to infer the comfort of particular exercises or motor tasks. This approach could also benefit from deep learning analysis.

## 6. Conclusions

The present study was formulated to explore the relationship between human manipulability and sEMG signals. According to the results of the performed exercises, elbow flexion produces a rise in manipulability indices, and wrist flexion and extension, on the contrary, are restrictive and less comfortable positions. The evaluated metrics for the sEMG give insights into how muscular effort is distributed during the exercises and could be useful to design movement sequences to target therapy goals according to muscle activity parameters, or to evaluate muscle performance in upper limb working tasks. However, the relationship between manipulability and muscular activity is not clear enough; therefore, a deeper analysis of the correlations between these parameters should be considered. A possible solution is to use deep learning algorithms to extract meaningful associations and provide insights into the application of these metrics in a particular set of exercises, to evaluate comfort in motor tasks or to assess different stages of rehabilitation programs.

## Figures and Tables

**Table 1:** Noraxon MiniDTS system details.

Sensor transmission range	20 m
Selectable low-pass cutoff	500/1000/1500 Hz
Wireless update rate	100 Hz
Selectable sample rate	3000/1500 Hz
Differential Input impedance	>10 Mohm
Baseline noise	5 uV RMS
Electronic gain	200
Overall gain	500

**Table 2:** DH parameters of the 6-DOF human arm model.

Joint	$\theta_{i}$	$\alpha_{i}$	$a_{i}$	$d_{i}$
1	q1	$\frac{\pi }{2}$	0	0
2	q3	$\frac{\pi }{2}$	0	0
3	q3	$\frac{\pi }{2}$	0	$-L_{1}$
4	q4	−$\frac{\pi }{2}$	0	0
5	q5	−$\frac{\pi }{2}$	0	$-L_{2}$
6	q6	0	$L_{3}$	0

## Data Availability

The data presented in this study are openly available in ScienceDB at https://doi.org/10.57760/sciencedb.01902 (accessed on 2 February 2023), as mentioned in [26].
